# Pretherapeutic Plasma Pro- and Anti- Inflammatory Mediators Are Related to High Risk of Oral Mucositis in Pediatric Patients with Acute Leukemia: A Prospective Cohort Study

**DOI:** 10.1371/journal.pone.0064918

**Published:** 2013-05-31

**Authors:** Ying Ye, Göran Carlsson, Monica Barr Agholme, Jenny Karlsson-Sjöberg, Tülay Yucel-Lindberg, Katrin Pütsep, Thomas Modéer

**Affiliations:** 1 Division of Pediatric Dentistry, Department of Dental Medicine, Karolinska Institutet, Huddinge, Sweden; 2 School and Hospital of Stomatology, Peking University, Beijing, China; 3 Childhood Cancer Research Unit, Department of Women’s and Children’s Health, Karolinska University Hospital, Karolinska Institutet, Stockholm, Sweden; 4 Department of Microbiology, Tumor and Cell Biology, Karolinska Institutet, Stockholm, Sweden; 5 Division of Periodontology, Department of Dental Medicine, Karolinska Institutet, Huddinge, Sweden; Ospedale Pediatrico Bambino Gesu’, Italy

## Abstract

**Objective:**

This prospective study evaluated clinical risk indicators as well as pro- and anti- inflammatory mediators at the time of malignancy diagnosis in relation to chemotherapy-related oral mucositis in pediatric population.

**Methods:**

Patients (n = 104) under 18 years of age with primary malignancies and undergoing chemotherapy were included. Potential risk indicators were analyzed using binary logistic regression with oral mucositis as the outcome. In a subgroup (n = 35), plasma samples at the time of malignancy diagnosis were analyzed for inflammatory cytokines and an antimicrobial protein pro-LL-37 (hCAP18).

**Results:**

In the multivariable model, type of malignancy diagnosis was significantly associated with oral mucositis, with highest risk of oral mucositis in patients with acute leukemia compared to those with lymphoma or solid tumors. At the time of malignancy diagnosis, plasma from patients with acute leukemia displayed higher concentrations (*P*<0.05) of IL-6, IL-8, IL-10, and TNF-α and lower levels of pro-LL-37 (*P*<0.001).

**Conclusions:**

The results imply that pretherapeutic high levels of inflammatory cytokines and low levels of pro-LL-37 in plasma might contribute to the high incidence of oral mucositis in patients with acute leukemia. These findings may add to our understanding of the predispositions to oral mucositis in children with malignancies.

## Introduction

Mucositis, which can involve the entire alimentary tract, is a major complication of cytostatic chemotherapy. Oral mucositis occurs in the mucosal lining of the oral cavity and mainly affects the non-keratinized epithelium. It may cause severe pain and bleeding, increase the risk of systemic infection, and further complicate anticancer treatment [1], [2].

The individual risk of oral mucositis during chemotherapy has been unpredictable, and there is a substantial variation among patients receiving identical treatment [3]. Within studies on pediatric patients, parameters including body weight prior to chemotherapy [4], blood type [5], underlying malignant disease [6], specific chemotherapy regimens or protocols [7], [8], serum creatinine level [4], blood methotrexate concentration [9], and neutropenia [4], [10], [11] have been suggested as risk factors for developing oral mucositis. However, the relative contribution of these risk factors in relation to mucositis is not clear, which makes it difficult to identify the patients at a higher risk of oral mucositis.

A five-stage mechanistic paradigm raised in the last decade describes the progression of mucositis as follows: initiation, signaling, amplification, ulceration and healing [12]. In this multifactorial event, the upregulation of pro-inflammatory cytokines, including IL-1β, IL-6, and TNF-α, was found to be critical in the initial phase [12]–[14]. Recently, it was revealed that the ability of monocytes to synthesize IL-10 before chemotherapy was inversely related to gastrointestinal mucositis [15]. However, the detailed pathobiology of mucositis has not been fully elucidated and the immunological predisposition to oral mucositis in terms of pretherapeutic peripheral pro- and anti- inflammatory cytokines has not been studied so far.

The impairment of innate or adaptive immunity in patients receiving cytostatic treatment may result in compromised protection of the mucosa against the oral microflora, which is highly diverse and complex [16]. We previously showed that the antimicrobial protein pro-LL-37, synthesized by myeloid cells in the bone marrow, is severely reduced in patients with severe congenital neutropenia, a condition of defective myelopoiesis [17]. In these patients, recurrent periodontal infection concurred with pronounced pro-LL-37 deficiency [18], [19]. In light of these findings, it is of interest to study the level of plasma pro-LL-37 in patients with malignancies and undergoing chemotherapy.

The current study was aimed to evaluate the potential risk indicators of oral mucositis in the pediatric cancer population. Furthermore, immunomodulatory parameters present prior to chemotherapy, which might predispose to mucosal destruction, including pro- and anti- inflammatory cytokines and pro-LL-37, were investigated at the time of malignancy diagnosis.

## Patients and Methods

### Patients

This study was designed as a prospective cohort study and was performed at the Pediatric Cancer Ward of Astrid Lindgren Children’s Hospital, Karolinska University Hospital, Stockholm, Sweden. Ethical permission was granted by the Regional Ethical Review Board in Stockholm, situated at Karolinska Institutet.

From November 2008 to December 2010, patients under 18 years of age with newly diagnosed malignancies who were referred to the Pediatric Cancer Ward of Astrid Lindgren Children’s Hospital were enrolled (n = 109). The exclusion criteria were as follows: (1) the treatment protocol did not consist of cytostatic drugs (n = 4) and (2) patients without national population registration (n = 1). A final tally of 104 patients was included in the present study. For the incidence calculation and risk indicator evaluation, the medical data were analyzed anonymously.

### Medical Records

For all included patients, data regarding age, gender, weight, height, body mass index (BMI), BMI-standard deviation scores (BMI-SDS) [20], diagnosis of cancer, and pre-existing chronic disease, were collected. Blood counts of neutrophils, lymphocytes, leukocytes and thrombocytes, and levels of hemoglobin and creatinine at the time of malignancy diagnosis were extracted from laboratory test reports. The results of blood antibody reactivity or PCR analysis for the presence of herpes simplex virus (HSV), Epstein-Barr virus (EBV), and cytomegalovirus (CMV) were also collected. In addition, oral tissues including the teeth, gingiva, and mucosa of each individual were examined by one of the authors. Decayed, missing or filled teeth (DMFT/dmft) were recorded based on clinical manifestations, and the gingival bleeding index (GBI) was estimated and categorized as <15% or >15%. For infants without teeth, the GBI was recorded as <15%. Oral hygiene instructions were given to both patients and parents before cytostatic treatment.

The grade of oral mucositis was evaluated using the World Health Organization (WHO) scoring system [21], which rates the oral toxic effect into five levels: grade 0, no change; grade 1, soreness/erythema; grade 2, erythema, ulcers, can eat solids; grade 3, ulcers, requires liquid diet only; grade 4, alimentation not possible. The evaluations were performed by two medical professionals with no inter-examiner difference. A WHO grade >1, which indicates ulcerative mucositis, was considered as occurrence of oral mucositis in the current study to avoid false positive diagnosis.

Potential treatment-related factors, including chemotherapeutic drugs and hematological adverse effects, were recorded. The cytostatic drugs included were methotrexate, doxorubicin/daunorubicin/idarubicin, cytarabine, cyclophosphamide/ifosfamide, actinomycin D, cisplatin/carboplatin, and etoposide. The hematological side effects included were neutropenia (absolute neutrophil count, ANC <0.5×10^9^/l), thrombocytopenia (thrombocytes <50×10^9^/l), and lymphopenia (lymphocyte <1.0×10^9^/l). For patients with oral mucositis, the data of cytostatic drugs in the latest chemotherapy course before every occasion of mucositis and occurrence of hematological adverse effects before mucositis were included in the statistical analysis. For patients without oral mucositis, the data of medications and hematological adverse effects during the entire treatment period was used in the statistical analysis. Any use of non-steroidal anti-inflammatory drugs was generally not allowed during the anticancer treatment due to the risk of bleeding.

### Plasma Sampling

Assent and written consent were obtained from 35 patients and their parents respectively to take peripheral blood at the time of malignancy diagnosis out of all the patients older than 4 years of age (n = 60). Coagulation of the blood was inhibited using EDTA. Following centrifugation, plasma was collected and stored at −20°C until analysis.

### Immunoblot and Multiplex Immunoassay

The level of pro-LL-37 was determined using immunoblotting. Plasma was dissolved in NuPAGE SDS sample buffer (Invitrogen, Sweden) and electrophoresed in 1.0 mm 4–12% NuPAGE Bis-Tris gels (Invitrogen) under reducing conditions. Immunoblotting was performed using a custom-designed rabbit anti-LL-37 antibody (Innovagen, Sweden) [18] that also detects pro-LL-37 and a goat anti-rabbit antibody (Dako, Sweden). Human serum (Sigma, Sweden) was used as the standard for the quantification of pro-LL-37. The results are expressed as percentage of the standard.

The concentrations of the cytokines IL-1β, IL-4, IL-6, IL-8, IL-10, IL-17, IFN-γ, and TNF-α were analyzed using multiplex immunoassays with LINCOplex human cytokine kit (Millipore, Sweden) and Bio-Plex 200 system (Bio-Rad, Sweden). The cytokine concentrations were expressed as pg/ml.

### Statistics

Data analysis was carried out using the statistical software package SPSS version 18.0. A binary logistic regression model was used to determine risk indicators for the outcome, oral mucositis. All independent variables with *P*<0.05 in the univariable analysis were included in a multivariable logistic model. A forward selection procedure was performed, with thresholds of *P*<0.05 for entry into the model and *P*>0.10 for removal from the model. Finally, variables remaining significant in the multivariable model were adjusted for the potential confounder methotrexate, which had shown significance in the univariable analysis. The odds ratio (OR) and 95% confidence interval (CI) were used as estimates of the effect of each variable. Oral mucositis free rates of different types of malignancy were estimated using the Kaplan-Meier method, and equality of the curves was analyzed using the log-rank test. The Mann-Whitney test with Bonferroni adjustment on *P*-values was used to compare pro-LL-37 and cytokine levels in patients with different malignancy diagnoses, which exclusively had shown significance in the multivariable logistic regression model. For all statistical methods used, the level of significance was accepted at α = 0.05.

## Results

Of the patients studied (n = 104), the mean age was 6.6 years, ranging from 12 days to 17 years. The subjects comprised 66 (63%) males and 38 (37%) females. The diagnoses of the patients are listed in Table 1.

**Table 1:** Diagnoses of the patients and frequencies of oral mucositis.

Diagnosis	All patients	Patients with oral mucositis
	n = 104	n = 59
	n (% of all patients)	n (% of each diagnosis)
Acute leukemia	40 (39%)	34 (85%)
ALL	33	27
AML	7	7
Lymphoma	14 (13%)	3 (21%)
Non-Hodgkin’s lymphoma	7	3
Hodgkin’s lymphoma^a^	7	0
Solid tumor	50 (48%)	22 (44%)
Brain tumor^a^	14	6
Renal tumor	8	0
Soft tissue sarcoma	8	5
Skeletal sarcoma	6	6
Retinoblastoma	6	0
Neuroblastoma	5	3
Hepatoblastoma	1	0
Pleuropulmonary blastoma	1	1
Nasopharyngeal carcinoma	1	1

ALL, acute lymphoblastic leukemia; AML, acute myeloid leukemia.

a Treatment protocols containing radiation do not involve orofacial region.

**Figure 1:**
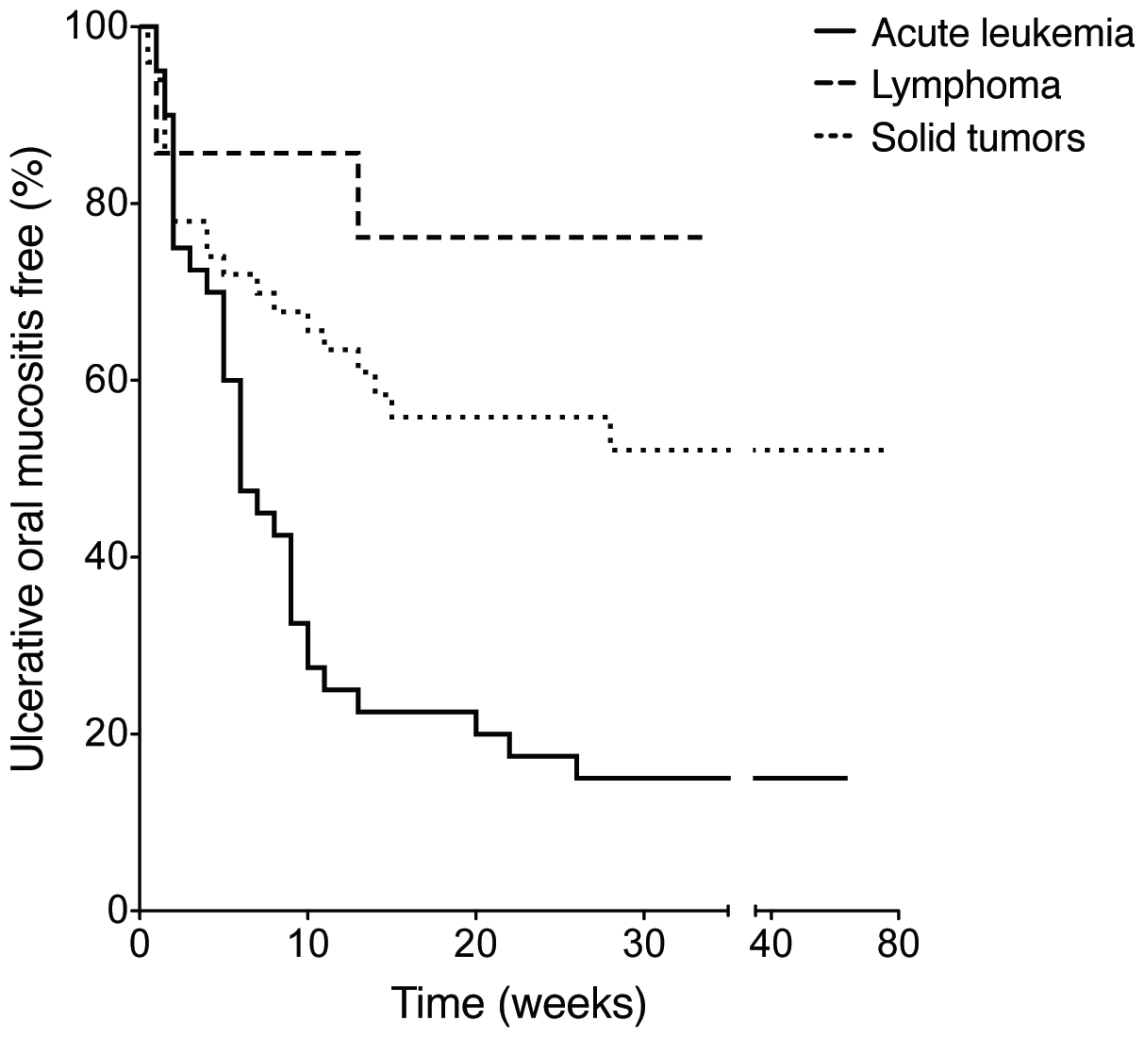
Kaplan-Meier curve of patients free from ulcerative oral mucositis with different types of malignancies. The horizontal axis starts from the beginning of the entire chemotherapy protocol. The oral mucositis (WHO grade >1) free rate is lower in patients with acute leukemia compared to those with lymphoma (*P* = 0.001) or solid tumors (*P* = 0.001). The oral mucositis free rates are not different between patients with lymphoma and those with solid tumors (*P* = 0.212).

During cancer treatment, 57% (59 out of 104) patients developed oral mucositis (WHO grade >1). The time from the start of the entire chemotherapy protocol to the occurrence of oral mucositis ranged from three days to 28 weeks, with a median value of six weeks in patients with acute leukemia, one week in lymphoma, and three weeks in solid tumors (Figure 1).

Based on all subjects studied, the univariable logistic regression analysis of each patient-related factor at the time of malignancy diagnosis and the outcome, oral mucositis, is listed in Table 2. Treatment-related factors during chemotherapy were also tested using univariable models (Table 3). All the covariates that showed significance were then included in a final multivariable model, in which the diagnosis of malignant disease (*P* = 0.001) was exclusively associated with occurrence of oral mucositis after the adjustment of methotrexate, with lower risks in lymphoma (OR = 0.054) and solid tumors (OR = 0.240), compared to acute leukemia (OR = 1 as reference) (Table 4). Further, taking into account of the dose-dependent toxicity of methotrexate, multivariable analysis adjusted for methotrexate dose was performed, in which malignancy diagnosis remained significant in association with oral mucositis (*P* = 0.001; lymphoma: OR = 0.054, 95% CI = 0.011–0.262; solid tumors: OR = 0.209, 95% CI = 0.068–0.641).

**Table 2:** Univariable logistic regression with patient-related factors at the time of malignancy diagnosis as independent variables and oral mucositis as the outcome in all patients (n = 104).

Variables	No oral mucositis	Oral mucositis	Univariable logistic regression	
	n = 45	n = 59	OR (95% CI)	*P-*value
	mean ± SD	mean ± SD		
Age (year)	5.9±5.7	7.1±5.3	1.045 (0.971–1.124)	0.238
Gender (M/F)	28/17	38/21	0.910 (0.407–2.035)	0.819
Weight (kg)	25.4±21.8	29.2±20.0	1.009 (0.990–1.029)	0.355
Height (cm)	109.5±39.4	122.0±35.4	1.009 (0.998–1.020)	0.095
BMI (kg/m^2^)	17.3±3.1	17.4±2.7	1.012 (0.882–1.161)	0.867
BMI-SDS	0.23±1.45	0.25±1.25	1.007 (0.751–1.349)	0.964
Oral variables				
DMFT (dmft)	0.6±1.7	0.4±1.1	0.924 (0.699–1.221)	0.578
GBI (≤15%/>15%)	42/3	55/4	1.018 (0.216–4.797)	0.982
Diagnosis				<0.001***
Acute leukemia	6	34	1	
Lymphoma	11	3	0.048 (0.010–0.225)	<0.001***
Solid tumors	28	22	0.139 (0.049–0.389)	<0.001***
Other chronic disease (yes/no)	10/35	17/42	1.417 (0.576–3.487)	0.449
Laboratory test				
Neutrophils (×10^9^/l)	5.2±4.8	3.6±4.5	0.925 (0.844–1.013)	0.093
Leukocyte (×10^9^/l)	31.6±135.3	43.4±106.6	1.001 (0.997–1.004)	0.621
Lymphocyte (×10^9^/l)	6.1±13.5	7.8±14.6	1.010 (0.978–1.043)	0.558
Thrombocyte (×10^9^/l)	364±306	192±157	0.995 (0.992–0.998)	<0.001***
Hemoglobin (g/l)	112±26	93±30	0.976 (0.960–0.991)	0.002**
Creatinine (µmol/l)	35±16	41±19	1.022 (0.998–1.047)	0.073
HSV^a^ (positive/negative)	10/31	16/41	1.210 (0.483–3.028)	0.684
EBV^a^ (positive/negative)	20/22	30/26	1.269 (0.569–2.829)	0.560
CMV^a^ (positive/negative)	20/22	25/34	0.809 (0.365–1.793)	0.601

BMI, body mass index; BMI-SDS, BMI-standard deviation scores; DMFT (dmft), decayed, missing or filled teeth; GBI, gingival bleeding index; HSV, herpes simplex virus; EBV, Epstein-Barr virus; CMV, cytomegalovirus; SD, standard deviation; OR, odds ratio; CI, confidence interval.

a Blood antibody reactivity or PCR analysis. Data of HSV in six patients, EBV in six patients, and CMV in three patients were not available.

** *P*<0.01,

*** *P*<0.001.

**Table 3:** Univariable logistic regression with treatment-related factors during chemotherapy as independent variables and oral mucositis as the outcome in all patients (n = 104).

Variables	No oral mucositis	Oral mucositis	Univariable logistic regression	
	n = 45	n = 59	OR (95% CI)	*P-*value
	n (%)	n (%)		
Cytostatic regimen				
Methotrexate	11 (24%)	34 (58%)	4.204 (1.790–9.872)	0.001**
Doxorubicin	27 (60%)	24 (41%)	0.457 (0.207–1.008)	0.052
Cytarabine	10 (22%)	18 (31%)	1.537 (0.628–3.761)	0.347
Cyclophosphamide	20 (44%)	14 (24%)	0.389 (0.168–0.901)	0.028^a^
Actinomycin D	11 (24%)	2 (3%)	0.108 (0.023–0.519)	0.005^a^
Cisplatin	19 (42%)	4 (7%)	0.100 (0.031–0.322)	<0.001^a^
Etoposide	22 (49%)	11 (19%)	0.240 (0.100–0.576)	0.001^a^
Hematological side effect				
Neutropenia (<0.5×10^9^/l)	33 (73%)	48 (81%)	1.587 (0.626–4.024)	0.331
Thrombocytopenia (<50×10^9^/l)	19 (42%)	36 (61%)	2.142 (0.972–4.718)	0.059
Lymphopenia (<1.0×10^9^/l)	28 (62%)	48 (81%)	2.649 (1.088–6.453)	0.032*

OR, odds ratio; CI, confidence interval.

a Variables with OR <1 were not included in multivariable analysis since cytostatic regimens can only be considered as risk indicators for oral mucositis.

* *P*<0.05,

** *P*<0.01.

**Table 4:** Multivariable logistic regression with oral mucositis as the outcome in all patients (n = 104).

Variables^a^	Unadjusted		Adjusted for methotrexate	
	OR (95% CI)	*P*-value	OR (95% CI)	*P*-value
Diagnosis		<0.001***		0.001**
Acute leukemia	1		1	
Lymphoma	0.048 (0.010–0.225)	<0.001***	0.054 (0.011–0.259)	<0.001***
Solid tumors	0.139 (0.049–0.389)	<0.001***	0.240 (0.075–0.765)	0.016*
Methotrexate^b^	–	0.052	2.879 (0.966–8.580)	0.058
Thrombocyte^b^	–	0.295	–	–
Hemoglobin^b^	–	0.845	–	–
Lymphopenia^b^	–	0.666	–	–

OR, odds ratio; CI, confidence interval.

a Variables that have significant difference in the univariable analysis (Table 2 and 3) are included.

b Variables that are removed from the logistic regression equation in the unadjusted model.

* *P*<0.05,

** *P*<0.01,

*** *P*<0.001.

The plasma parameters from the subgroup (n = 35), from which we obtained plasma samples at the time of malignancy diagnosis, were compared across malignancy type (Table 5), which was shown as the primary risk indicator in the multivariable model based on all patients. This subgroup had similar distributions in age (between 4–18 years, *P* = 0.896), gender (*P* = 0.176), diagnosis (*P* = 0.310), and oral mucositis occurrence (*P* = 0.249) as the entire studied group (n = 104). Cases of lymphoma (n = 7) and solid tumors (n = 13) were combined since there was no inter-group difference in any of the plasma parameters analyzed. Compared to lymphoma or solid tumors (n = 20), acute leukemia subjects (n = 15) displayed significantly higher concentrations (*P*<0.05) of IL-6, IL-8, IL-10, and TNF-α and lower levels of pro-LL-37 (*P*<0.001). In order to further illustrate the relation between pretherapeutic plasma parameters and the outcome oral mucositis, comparisons were also performed between patients that later developed oral mucositis (n = 24, including 15 of acute leukemia, 9 of lymphoma or solid tumors) and those that did not (n = 11, which are all with lymphoma or solid tumors). Higher concentrations of IL-6 (*P*<0.001), IL-8 (*P* = 0.009), and IFN-γ (*P* = 0.027) as well as lower levels of pro-LL-37 (*P* = 0.009) were demonstrated in patients that developed oral mucositis compared to those that did not.

**Table 5:** Plasma pro-LL-37 (%)^a^ and cytokines (pg/ml) at the time of malignancy diagnosis in patients (n = 35) with different malignancy diagnosis.

Variables	Acute leukemia	Lymphoma or solid tumors^c^	*P*-value^d^
	n = 15^b^	n = 20	
	median (range)	median (range)	
Pro-LL-37	1 (0–26)	49 (2–314)	<0.001***
IL-1β	1.7 (0.0–5.0)	0.8 (0.0–11.0)	1.000
IL-4	2.2 (0.0–61.0)	0.5 (0.0–7.5)	1.000
IL-6	10.9 (0.0–89.3)	0.8 (0.0–22.1)	0.009**
IL-8	15.6 (6.5–51.4)	1.7 (1.7–13.7)	<0.001***
IL-10	14.3 (1.8–1161.9)	3.7 (0.7–13.9)	0.018*
IL-17	3.1 (0.0–42.7)	0.0 (0.0–24.9)	0.360
IFN-γ	5.6 (0.0–63.5)	0.4 (0.0–61.8)	0.378
TNF-α	6.8 (0.0–59.8)	0.0 (0.0–7.0)	0.018*

a Percentage of the standard.

b Cytokine concentrations were tested on 14 patients.

c There was no plasma parameter different between lymphoma and solid tumors.

d Mann-Whitney test with *P*-value adjusted with Bonferroni method.

* *P*<0.05;

** *P*<0.01;

*** *P*<0.001.

In order to further investigate the mucositis risk in patients of each malignancy group, we then performed logistic regression on stratified data by malignancy diagnosis. Lymphoma cases were excluded due to a limited patient number (n = 14). In the patients with acute leukemia (n = 40), there was no variable that showed significant association with oral mucositis in the univariable analysis. In the patients with solid tumors (n = 50), age (*P* = 0.015), weight (*P* = 0.030), height (*P* = 0.008), lymphocyte count (*P* = 0.024), and methotrexate (*P* = 0.023) showed significance in the univariable models. Interestingly, in the final multivariable model based on patients with solid tumors, lymphocyte count (*P* = 0.017, OR = 0.353, 95% CI = 0.149–0.833) was exclusively associated with the occurrence of oral mucositis after the adjustment of methotrexate.

## Discussion

Early identification of the patients with high risk of oral mucositis and understanding the predispositions are of clinical importance. In the current study, we showed that at the time of malignancy diagnosis, patients with acute leukemia, who had the highest risk of oral mucositis, showed high concentrations of both pro- and anti- inflammatory cytokines and low levels of pro-LL-37 in the plasma.

In order to identify the most essential clinical indicator(s) of oral mucositis, variables present before chemotherapy and those arising during chemotherapy were evaluated. In the multivariable logistic model based on all the patients, the malignancy diagnosis exclusively showed significance after the adjustment of the potential confounder methotrexate. It is noteworthy that in the current study, individual cytostatic drugs, instead of different chemotherapy protocols, were included as covariates in an attempt to better evaluate the direct association between cytostatic regimens and the outcome, oral mucositis. The adjustment of the potential confounder methotrexate did not affect the significance of malignancy diagnosis in the multivariable model, which indicates that the association of acute leukemia and oral mucositis might be far more complex than the direct cytostatic effect of the intensive chemotherapy protocol applied.

The levels of pro- and anti- inflammatory mediators at the time of malignancy diagnosis were compared across malignancy types. The plasma from patients with acute leukemia displayed significantly higher concentrations of both pro-inflammatory cytokines (IL-6, IL-8, and TNF-α) and the anti-inflammatory cytokine (IL-10). This finding suggests a significantly elevated inflammatory status in patients with acute leukemia before the start of chemotherapy. It has long been known that the upregulation of pro-inflammatory cytokines following cytostatic treatment is essential in promoting oral mucositis during cancer therapy [12], [14], [22], [23]. Therefore, the higher concentrations of pretherapeutic inflammatory cytokines could reasonably be expected to contribute to the high risk of oral mucositis in patients with acute leukemia. In addition, in the comparison between patients with different outcome in terms of oral mucositis, patients that later developed oral mucositis were found to have higher concentrations of pro-inflammatory cytokines (IL-6, IL-8, and IFN-γ) at the time of cancer diagnosis, which could be largely explained by the high proportion of cases with acute leukemia in this group (15 out of 24).

The plasma levels of pro-LL-37 at the time of malignancy diagnosis were lower in patients with acute leukemia compared to those with lymphoma or solid tumors. Moreover, the pretherapeutic levels of pro-LL-37 were lower in patients that later developed oral mucositis, which coincided with the higher concentrations of pro-inflammatory cytokines. The antimicrobial protein pro-LL-37 (also named hCAP18) is the proform of the single human cathelicidin type of antimicrobial peptide LL-37 [24], [25] and is detectable in the plasma [26]. Oral infections and inflammations especially chronic periodontitis have been associated to patients with deficiencies in pro-LL-37 [19], [27], which underscores the protective role of pro-LL-37 in oral health. Thus, a pretherapeutic low level of pro-LL-37 may render the oral mucosa more vulnerable to the destruction caused by commensal or pathological oral bacteria.

Interestingly, in the logistic regression analysis performed on stratified data according to malignancy diagnosis, the low lymphocyte count at the time of malignancy diagnosis was found to be an indicator of oral mucositis in patients with solid tumors. This may reflect an impaired immune control of the resident mucosal microflora. It has been reported that patients with improved lymphocyte recovery after chemotherapy display decreased severity of gastrointestinal mucositis [28], while our results demonstrate that lymphocyte levels before chemotherapy may also be important in the pathogenesis of oral mucositis in patients with solid tumors. In the patients with leukemia, there was no additional variable associated to oral mucositis. This may due to the high frequency (85%) of oral mucositis in these patients, which makes the identification of any risk indicator difficult.

Chemotherapy generally affects the immune system by reducing the number of leukocytes. The role of neutropenia has been controversial with regard to the development of oral mucositis [11], [29], [30]. In the current study, the occurrence of neutropenia was evaluated strictly only before the time point of oral mucositis appearance. Neither neutrophil counts at the time of malignancy diagnosis nor the occurrence of neutropenia was found to be associated with oral mucositis, which suggested that neutropenia might only be a concomitant side effect rather than a risk indicator of oral mucositis as previously suggested [4]. Further studies will be needed to demonstrate the role of other immune components and/or local epithelial defense in the development of oral mucositis.

In conclusion, the current study shows that at the time of malignancy diagnosis, patients with acute leukemia, who had highest risk of oral mucositis, presented high concentrations of inflammatory cytokines and low levels of pro-LL-37 in the plasma. This pretherapeutic elevated inflammatory state might predispose the patients to oral mucositis. Our novel findings could contribute to the early identification of patients at high risk of oral mucositis and also benefit the prophylactic management in an effort to decrease or avoid severe oral mucositis in pediatric patients with malignancies.
